# A rare image of spontaneous enterocutaneous fistula in morbidly obese patient with metabolic syndrome

**DOI:** 10.11604/pamj.2015.20.83.5814

**Published:** 2015-01-29

**Authors:** Ballah Akawu Denue, Salihu Aliyu Kwayabura

**Affiliations:** 1 Department of Medicine, University of Maiduguri Teaching Hospital, Bama Road, Maiduguri, Borno State, Nigeria

**Keywords:** Spontaneous enterocutaneous fistula, morbid obesity, metabolic syndrome

## Image in medicine

32 year old lady, a morbidly obese grand multipara developed spontaneous enterocutaneous fistula. Wound around right para-umblical region started as boil, it ruptured and started discharging purulent pus associated fever, colicky central abdominal pain, nausea and non projectile vomiting of recently ingested fluid. One week to presentation she started discharging feacal material from the wound site, she had intravenous ceftriazone 1gram twice daily and intravenous metronidazole 500 mg eight hourly for 5 days at referring hospital. On examination, she was morbidly obese (BMI = 45kg/m^2^), febrile, pale (Haematocrit = 9g/dl), anicteric, no pedal edema. She had elevated BP= 170/80mmHg, E/U/Cr was normal, elevated uric acid was 9.0 mg (4.0 - 6.5)mg, Fasting lipid profile (Total Cholesterol 206 mmol/l, Triglyceride 157 mmol/l, HDL 39 mmol/l, LDL 172 mmol/l), Fasting blood glucose of [6.9mg/dl(2.5 - 6). Patient is a known dyspeptic and Diabetes mellitus. Spontaneous fistula is rare in clinical practice and often complicated by malnutrition, fluid and electrolyte imbalance, anemia and sepsis. The Triad of sepsis, malnutrition, and fluid/electrolytes disturbance is associated with high mortality. The index case presented with anemia and sepsis with no significant weight loss. In view of rarity of this presentation, an image of spontaneous enterocutaneous fistula with peri umblical sepsis in a young lady with metabolic syndrome is hereby presented.

**Figure 1:**
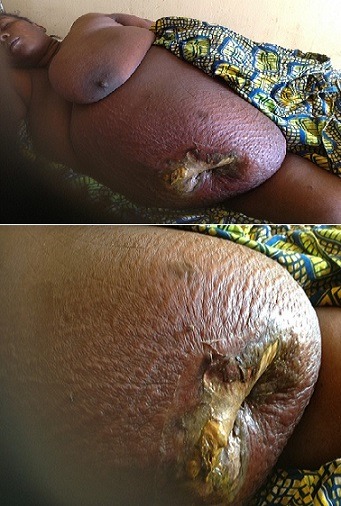
Spontaneous enterocutaneous fistula with periumblical sepsis in a lady with metabolic syndrome

